# A case report of recessive restrictive cardiomyopathy caused by a novel mutation in cardiac troponin I (*TNNI3*)

**DOI:** 10.1186/s12881-019-0793-z

**Published:** 2019-04-05

**Authors:** Malena P. Pantou, Polyxeni Gourzi, Aggeliki Gkouziouta, Iakovos Armenis, Loukas Kaklamanis, Christianna Zygouri, Pantelis Constantoulakis, Stamatis Adamopoulos, Dimitrios Degiannis

**Affiliations:** 1 Molecular Immunopathology and Histocompatibility Unit, Division of Genetics, Onassis Cardiac Surgery Center, Syggrou Av, 356, 176 74 Athens, Greece; 2 Heart Failure, MCS and Transplant Unit, Onassis Cardiac Surgery Center, Athens, Greece; 3 Department of Pathology, Onassis Cardiac Surgery Center, Athens, Greece; 4 Department of Molecular Genetics, BioAnalytica-Genotypes S.A, Athens, Greece

**Keywords:** *TNNI3*, Restrictive cardiomyopathy, Autosomal recessive, Cardiac troponin I, Mutation, Case report

## Abstract

**Background:**

Restrictive cardiomyopathy is a rare cardiac disease, for which several genes including *TNNT2*, *MYPN*, *FLNC* and *TNNI3* have been associated with its familial form.

**Case presentation:**

Here we describe a female proband with a severely manifested restrictive phenotype leading to heart transplantation at the age of 41, who was found homozygous for the novel *TNNI3* mutation: NM_000363.4:c.586G > C, p.(Asp196His). Her parents were third-degree cousins originating from a small village and although they were found heterozygous for the same variant they displayed no symptoms of the disease. Her older sister who was also found heterozygous was asymptomatic. Her twin sister and her brother who were homozygous for the same variant displayed a restrictive and a hypertrophic phenotype, respectively. Their children are all carriers of the mutation and remain asymptomatic until the age of 21.

**Conclusion:**

These observations point to a recessive mode of inheritance reported for the first time for this combination of gene/disease.

## Background

Restrictive cardiomyopathy (RCM) is a rare cardiac disorder that manifests primarily as an abnormality of diastolic filling, which is also reported as a reduced ventricular compliance or increased stiffness, leading to reduced diastolic volume with preserved systolic function [1]. Several studies have confirmed that RCM can be inherited in a familial manner (MIM#PS115210) and mutations in several genes have been associated with the restrictive phenotype i.e. *TNNT2* (MIM*191045) [2], *MYPN* (MIM*608517) [3] and *FLNC* (MIM*102565) [4]. In 2003, Mogensen et al. [5] showed a causal relationship between cardiac troponin I genetic abnormalities and the manifestation of the disease after studying several heterozygous patients diagnosed with idiopathic restrictive cardiomyopathy, including one large family in which affected individuals presented with either restrictive or hypertrophic cardiomyopathy (HCM).

Cardiac troponin serves as a sensor of the intracellular Ca^2+^ levels and regulates the interaction between the thick and thin filaments during muscle contraction. It consists of three separate subunits: troponin C, troponin T and troponin I (cTnI; *TNNI3*), the latter being the inhibitory subunit of the complex, primarily functioning to prevent actin and myosin from interacting in the absence of Ca^2+^. Mutations in the troponin complex introduce alterations in Ca^2+^ affinity and protein-protein interactions, which may ultimately lead to the development of cardiomyopathy.

We describe here a female patient with a severely manifested phenotype of RCM leading to heart transplantation at the age of 41, attributed to an homozygous mutation in the gene encoding the cTnI (*TNNI3*). In her family all heterozygous carriers were found asymptomatic, while her homozygous sister and brother displayed restrictive and hypertrophic phenotype, respectively, pointing to a recessive mode of inheritance reported for the first time for this combination of gene and disease.

## Case presentation

### Patients

**Fig. 1:**
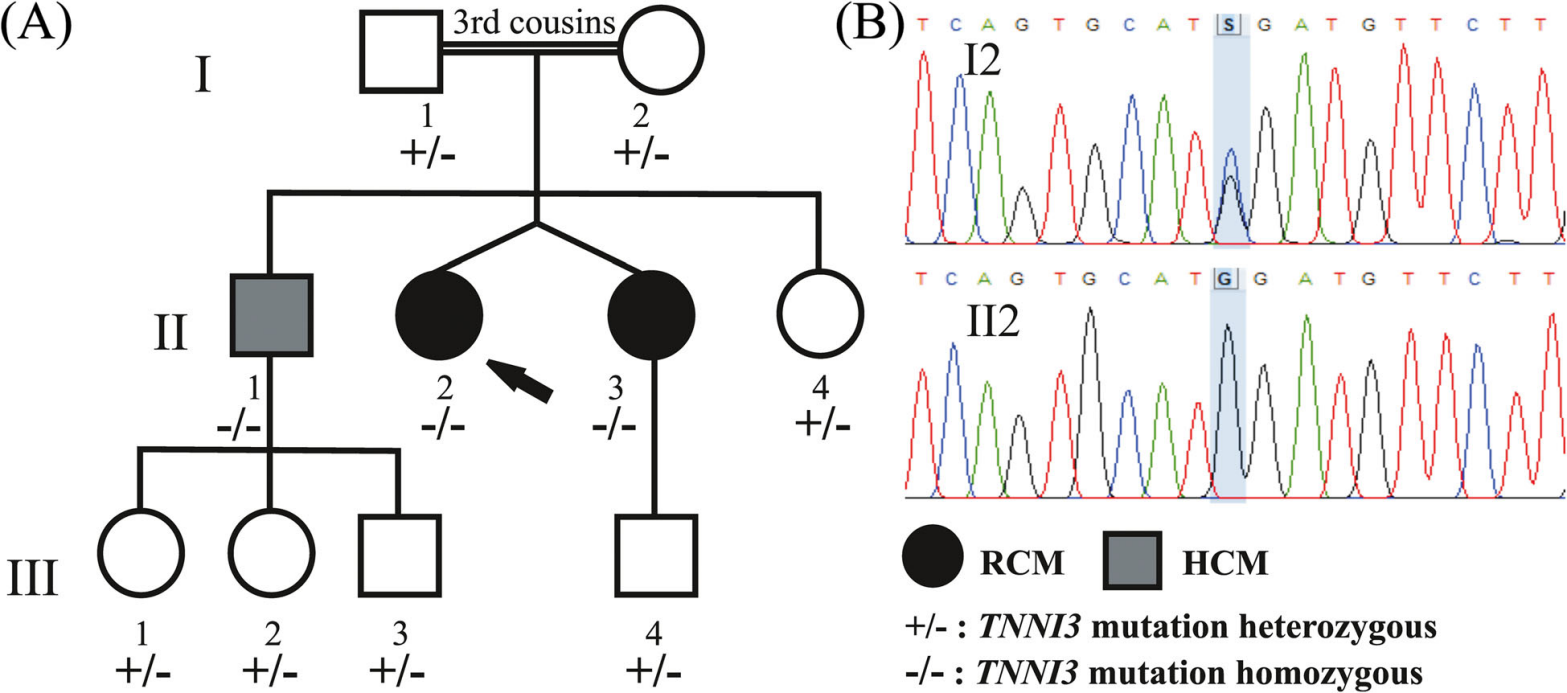
**a** The family pedigree is depicted. The proband (shown by arrow, II2) and her dizygotic twin sister (II3) were homozygous for the NM_000363.4:c.586G > C variant and displayed the phenotype of restrictive cardiomyopathy (RCM). Their brother (II1) also homozygous for the same variant displayed the phenotype of hypertrophic cardiomyopathy (HCM). Their children, with age range from 5 to 21 years old (III1: 5, III2: 13, III3 and III4: 21), remain unaffected along with the proband’s parents who were found heterozygous for the same mutation. Circle = female. Square = male. Filled symbols = affected individuals. **b** Electropherogram of the involved sequence fragment of the *TNNI3* for the members of the family. The detected mutation is highlighted

**Fig. 2:**
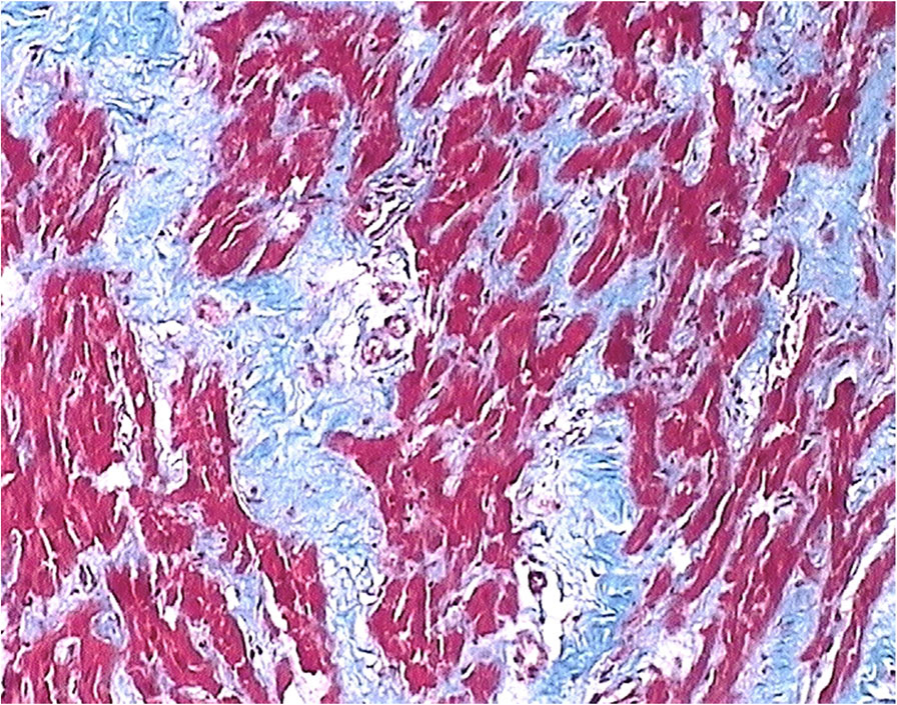
The histology of the affected myocardium revealed multiple foci of intestitial and pericellurar fibrosis with features of non-specific myocytic hypertrophy. There was no evidence of inflammation or deposition of amyloid. (trichrome stain Χ100)

The proband (patient II2, Fig. 1a) was referred to our hospital at the age of 41 due to progressive dyspnea on exertion over the last 3 months. Her medical history was remarkable for ischemic stroke attributed to paroxysmal atrial fibrillation. She was on oral anticoagulation (acenocumarol). Her electrocardiogram showed atrial fibrillation and her echocardiographic study revealed normal left ventricular (LV) dimensions with borderline contractile function (left ventricular ejection fraction [LVEF] 50%), severe biatrial dilatation and mild mitral regurgitation. Transmitral diastolic flow revealed monophasic flow (atrial fibrillation) with normal average maximal velocity (E) but shortened deceleration time (DT 110 ms), a finding consistent with restrictive LV filling (Table 1). Right heart catheterization demonstrated increased left atrial pressure (estimated through pulmonary capillary wedge pressure), pulmonary hypertension and restrictive diastolic filling pattern with characteristic dip-plateau morphology, confirming echocardiographic findings. Attempts to restore sinus rhythm were unsuccessful. Despite receiving appropriate treatment (metoprolol tartate, acenocoumarol, furosemide, amiloride) and achieving adequate rate control, the patient deteriorated during the following year and a new echocardiographic study revealed worsened LV systolic function (LVEF 35%, LV end-systolic diameter 34 mm), similar left and right atrial volumes, moderate mitral and tricuspid regurgitation and pericardial effusion, while right ventricular function was also impaired. Right heart catheterization confirmed hemodynamic deterioration and the patient was admitted to our center’s heart transplantation list. Due to further clinical aggravation, a pulsatile biventricular assist device was implanted as a bridge to transplant and two months later, successful heart transplantation was performed. The histology of the native heart revealed multiple foci of intestitial and pericellurar fibrosis with features of non-specific myocytic hypertrophy (Fig. 2).

**Table 1:** Clinical, Electrocardiographic, and Doppler Echocardiographic Data of the proband and her family members. Approximate normal ranges of echocardiographic data according to published sources are shown in brackets

Member of the family	TNNI3 genetic status	Current age/sex	Age at symptom onset-examination	Height (m)/ BSA (m^2^)	ECG, Arrhythmia	LVEDD (mm)	IVSd (mm)	PWd (mm)	LVESD (mm)	LVEF %	LAVi (ml/m^2^)	RAVi (ml/m^2^)	E (cm/s)	A (cm/s)	E/A	DT (ms)	Accompanying disease/habits (therapy)
II1	Hom	49 M	42	1.75/2.01	SR, incomplete RBBB, LV strain	47 [42–58]	**12** [6–10]	10 [6–10]	30 [25–40]	60 [52–72]	**53.7** [16–34]	38.8 [11–39]	54 [40–104]	71 [32–92]	0.77 [0.60–1.84]	190 [81–293]	HCM, NYHA II, presyncope, Dyslipidemia, smoker (acetylsalicylic acid, rosuvastatin)
Proband II2	Hom	52F	41	1.60/1.49	AF, LVH, LV strain	45 [38–52]	7 [6–9]	7 [6–9]	31 [22–35]	**50** [54–74]	**127.5** [16–34]	**100.7** [9–33]	60 [43–111]	na (AF)	na (AF)	110 [109–268]	RCM, NYHA III-IV (acenocumarol, metoprolol tartate, furosemide, amiloride)
II3	Hom	52F	45	1.60/1.59	SR, incomplete RBBB, LV strain	44 [38–52]	8 [6–9]	8 [6–9]	30 [22–35]	55 [54–74]	**94.3** [16–34]	47.2 [9–33]	65 [43–111]	**24** [35–91]	**2.71** [0.4–2.12]	104 [109–268]	RCM, NYHA II (furosemide)
II4	Het	55F		1.65/1.79	SR	42 [38–52]	7 [6–9]	7 [6–9]	26 [22–35]	55 [54–74]	33.5 [16–34]	27.9 [9–33]	63 [43–111]	75 [35–91]	0.87 [0.4–2.12]	**300** [109–268]	Asymptomatic
I1	Het	75 M		1.70/1.94	SR	46 [42–58]	10 [6–10]	10 [6–10]	27 [25–40]	65 [52–72]	23.2 [16–34]	23.7 [11–39]	60 [37–97]	75 [41–105]	0.80 [0.42–1.50]	280 [78–357]	Asymptomatic Dyslipidemia (rosuvastatin)
I2	Het	72F		1.70/2.00	SR	41 [38–52]	**10** [6–9]	9 [6–9]	26 [22–35]	55 [54–74]	**35** [16–34]	27.9 [9–33]	50 [38–106]	75 [44–108]	0.67 [0.37–1.61]	310 [90–313]	Asymptomatic Increased apical trabeculations, AH, Dyslipidemia (olmesartan, rosuvastatin, acetylsalicylic acid)
III2	Het	13F		1.61/1.45	SR	48 [38–52]	7 [6–10]	6 [6–10]	27 [22–35]	60 [54–74]	33.0 [18–34]	14.9^a^ [9.5–19.3]	100 [50–118]	70 [27–75]	1.43 [0.68–2.76]	190 [97–257]	Asymptomatic
III3	Het	21 M		1.78/1.97	SR	50 [42–58]	8 [6–10]	9 [6–10]	34 [25–40]	55 [52–72]	25.4 [16–34]	22.3 [11–39]	80 [51–107]	45 [24–76]	1.78 [0.65–2.73]	195 [87–273]	Asymptomatic

^a^ for pediatric patient III2 Right Atrial Area is documented instead of RAVi

A: maximal velocity of the late atrial component of transmitral blood flow, AF: Atrial Fibrillation, AH: Arterial Hypertension, BSA: Body Surface Area calculated with Mosteller formula, DT: Deceleration Timeof early transmitral flow (time from maximal velocity to zero), E: maximal early transmitral blood flow velocity, ECG: ElectroCardioGram, HCM: Hypertrophic Cardiomyopathy, Het: heterozygous, Hom: homozygous, IVSd: Interventricular Septum Diameter, LAVi: Left Atrial Volume indexed to BSA, LV: Left Ventricle, LVEDD: Left Ventricular End Diastolic Diameter, LVEF: LV Ejection Fraction, LVESD: Left Ventricular End Systolic Diameter, LVH: Left Ventricular Hypertrophy, na: not applicable, NYHA: New York Heart Association class, PWd: Posterior Wall Diameter, RAVi: Right Atrial Volume indexed to BSA, RBBB: Right Bundle Branch Block, RCM: Restrictive Cardiomyopathy, SR: Sinus Rhythm

Abnormal values are shown in bold letters

The proband’s twin sister (patient II3), although initially asymptomatic, underwent screening exercise stress test and cardiac ultrasound at the age of 43. The stress test, despite LV strain findings on the resting electrocardiogram, was substantially negative for heart ischemia. The cardiac ultrasound revealed normal LV dimensions (interventricular septum 8 mm, posterior wall 8 mm, LV end-diastolic diameter 44 mm, LV end-systolic diameter 30 mm), systolic function (LVEF 55%) and left atrial enlargement (left atrial volume 94.3 ml/m^2^). Doppler echocardiography showed restrictive pattern of diastolic transmitral flow (early diastolic transmitral blood flow velocity E higher than late atrial contraction-induced velocity A, E/A > 2, decreased deceleration time of E: DT 120 ms), and increased right ventricular systolic pressure (55 mmHg), as well as mild mitral and tricuspid valve regurgitation. At the age of 45, she started complaining for exertional dyspnea. A new cardiac ultrasound confirmed the previously reported findings with similar E/A ratio and even lower DT (104 ms, Table 1). She underwent right heart catheterization, which confirmed restrictive diastolic filling pattern and pulmonary hypertension. As a result, the diagnosis of a form of restrictive cardiomyopathy was established and the patient was managed medically (low doses of furosemide per os) remaining stable with mild symptoms. Recently, at the age of 51, her symptoms were aggravated due to the occurrence of atrial fibrillation.

Their brother (patient II1) was admitted to hospital at the age of 42 due to recurrent pre-syncope and exertional dyspnea. His electrocardiogram revealed sinus rhythm with incomplete Right Bundle Branch Block and nonspecific repolarization abnormalities. His echocardiographic study was remarkable for asymmetric hypertrophy of the interventricular septum and papillary muscles, left atrial enlargement (milder than his affected sisters’), mild mitral regurgitation and diastolic dysfunction without restrictive pattern (E < A, normal DT), while LV size and function were normal (Table 1). Doppler velocity measurements in the LV outflow tract were normal (absence of outflow tract obstruction). The above findings were consistent with HCM. The patient showed no significant epicardial vessel stenoses, but acetylsalicylic acid and rosuvastatin were initiated. He also underwent 24-h ambulatory heart rhythm recording, which was negative for ventricular arrhythmias. The patient remains stable with minimal symptoms and his cardiac ultrasound has not changed remarkably.

His parents (I1 and I2) along with one of his sisters (II4) remain asymptomatic. The echocardiogram of the proband’s mother (patient I2) presented prominent apical trabeculations, but her electrocardiogram was essentially normal. Similarly all of the children (III1–4) of patients II1 and II3 are asymptomatic. The youngest child (III1) is 5 years old and therefore has not been clinically evaluated yet. The clinical data of children III2 (age 13) and III3 (age 21) are listed in Table 1 and for the child III4 (age 21) although reported asymptomatic no data are available as he is clinically monitored in another medical center.

### Genetic analysis

The molecular basis of the disease was identified for the proband with next generation sequencing technology, using Illumina’s Trusight Cardio sequencing panel, covering 174 genes clinically relevant to cardiac diseases. Alignment, quality filtering, variant calling, and variant annotation were performed using in parallel the standard MiSeq Reporter (Illumina) and the Sophia Genetics pipeline. The variant calling files were filtered using VariantStudio Data Analysis Software (Illumina, San Diego, CA) and the Sophia Genetics DDM platform and the detected variants were characterized according to the recommendations of the American College of Medical Genetics and Genomics (ACMG) [6]. All benign or likely benign variants were filtered out and the retained variants were then evaluated according to the relevance of the gene to the observed phenotype resulting in one plausible candidate variant which was located in the *TNNI3* gene: NM_000363.4:c.586G > C, p.(Asp196His). The variant (https://databases.lovd.nl/shared/individuals/00204288) was novel and characterized as likely pathogenic (according to ACMG criteria). It was absent from the population databases of Exome Sequencing Project and Genome Aggregation Database (PM2 criterion) and pathogenicity predictions were all in favor of a damaging effect of this amino acid substitution (PP3 criterion) (SIFT: 0.001, PolyPhen-2 HumVar: 0.948, Mutation Taster: 1.000 and CADD v1.3: 32). The variant affected a highly conserved nucleotide (phyloP: 4.56 [− 14.1;6.4]) and a highly conserved amino acid (Consurf: 8, [1–9]) and was located in a region where mutations of nearby residues support its functional importance [7], while missense variants in *TNNI3* are a common mechanism of RCM (PP2 criterion). Additionally, another amino-acid missense variant at this position, p.Asp196Asn, is classified as likely pathogenic using ACMG criteria (PM5 criterion). Surprisingly, the proband was found homozygous for this variant but it was the second child of parents that are third cousins and originate from the same small mountainous village in Peloponnese, Greece (Fig. 1). The family of the proband underwent *TNNI3* genetic testing by targeted Sanger sequencing for the detection of the variant. Her twin sister and her brother were found homozygous for the variant, while both parents and the older sister were found heterozygous (Fig. 1).

## Discussion and conclusions

The C-terminal of troponin I (amino acids 184–210 in human cardiac TnI) is the most conserved structure of the molecule and interacts with tropomyosin in a calcium-regulated manner, suggesting a functional role. Despite its degree of conservation this region is highly flexible with no stable secondary structure [8] supporting the hypothesis that it is a dynamic structure in the troponin functions. The end segment of cTnI (amino acids 190–210) has been shown to play a role in the stabilization of tropomyosin in the actin filament upon Ca^2+^ activation [9]. In vivo data support that mutations in this region cause myofibril Ca^2+^ hypersensitivity and subsequent impaired relaxation, which is the main manifestation of RCM [10], suggesting that this region may constitute a mutational hotspot. Several variants located in this C-terminus end segment of the protein have been associated with HCM, while three of them (p.Asp190Gly, p.Arg192His and p.Arg204His) have been also reported to be associated with RCM as well [11]. In fact, the genetic investigation of a large family has revealed that carriers of the same variant (p.Asp190Gly) displayed considerable phenotypic heterogeneity with most of them fulfilling the HCM diagnostic criteria, while some of them were diagnosed with RCM [5]. Additionally, variants affecting the same amino acid position have been reported to confer to either the same (i.e. p.Arg192His and p.Arg192Cys are both associated with RCM) [5, 12] or different phenotypes (i.e. p.Leu144Gln is associated to RCM while p.Leu144Pro is associated to HCM) [5, 13].

Genetic analysis of our proband that presented a severe restrictive cardiomyopathy phenotype leading to heart transplantation at the age of 41, revealed a novel *TNNI3* variant at homozygous state: NM_000363.4:c.586G > C, p.(Asp196His). A different variant located at the same amino acid position i.e. NM_000363.4:c.586G > A, p.(Asp196Asn) has been repeatedly associated with HCM [14–16], while recently the variant p.Asp196Gly was detected in a patient diagnosed with atrial fibrillation [17]. The parents of our proband are third-degree cousins originating from a small mountainous village in Peloponnese, Greece and are asymptomatic carriers. Homozygous *TNNI3* mutations have been associated with recessive forms of DCM [18] and HCM [19] but not RCM so far.

Restrictive cardiomyopathy is considered to be inherited in an autosomal dominant way, although recently Ploski et al. [20] have reported that *TNNC1* is a likely novel gene for autosomal recessive restrictive cardiomyopathy. In this family, all heterozygous carriers were asymptomatic pointing to a recessive form of the disease, but it is important to stress out that in families with restrictive cardiomyopathy inherited as dominant trait, penetrance is reduced and age dependent, so the phenotype of generation III may have not reached maturity (Fig. 1) [11]. Nevertheless, this novel variant seems less pathogenic compared to the other amino acids substitutions observed at the same position. This may reflect subtle functional differences of the different amino acids, as it has been shown for mutations at position 204, where p.Arg204His mutation affected interaction of cTnI with troponin T and troponin C, while p.Arg204Cys affected interaction with troponin C but not troponin T and both produced increased Ca^2+^ sensitivity [21]. Moreover, one heterozygous carrier (I2), despite being asymptomatic with normal LV systolic and diastolic function, exhibits an echocardiographic LV morphology resembling noncompaction cardiomyopathy. As *TNNI3* mutations have been reported in noncompaction cardiomyopathy patients [22], a partially expressed phenotype even in heterozygous state cannot be firmly excluded. Of course, increased apical trabeculations may be a random finding unrelated to the specific mutation. Furthermore, the observed phenotypic heterogeneity in the three homozygous patients support the action of modifying genetic and/or environmental mechanisms.

## Abbreviations

ACMG: American College of Medical Genetics and Genomics
HCM: Hypertrophic cardiomyopathy
LV: Left ventricular
LVEF: Left ventricular ejection fraction
RCM: Restrictive cardiomyopathy
